# Chronic Myelogenous Leukemia Presenting with Facial Nerve Palsy in an Infant

**Published:** 2018

**Authors:** Ali KHAJEH, Ghasem MIRI ALIABAD, Afshin FAYYAZI, Gholamreza SOLEIMANI, Reza KEIKHA

**Affiliations:** 1 Department of Pediatrics, Children and Adolescent Health Research Center, Zahedan University of Medical Sciences, Zahedan, Iran; 2 Department of Pediatrics, Hamedan University of Medical Sciences, Hamedan, Iran; 3 Department of Biotechnology, Zahedan University of Medical Sciences, Zahedan, Iran

**Keywords:** Chronic myelogenous leukemia, Childhood, CNS involvement, Cranial nerve palsy

## Abstract

When a child presents with cranial nerve palsy and a bulging fontanel, a pediatric neurologist is often consulted to determine the cause of increased intracranial pressure. This report describes an infant with chronic myelogenous leukemia (CML) referred to Ali-bin-Abitaleb Hospital, Zahedan, eastern Iran in 2013 who presented with seventh cranial nerve palsy and bulging fontanel. Chromosomal analysis and peripheral blood smear confirmed the diagnosis of CML.

## Introduction

CML is a disease that predominantly affects middle-aged adults, with a peak incidence during the fourth and fifth decades of life. Most pediatric CML cases are diagnosed after 4 yr of age. No racial or gender differences exist for most pediatric cases (1). CML in infancy is uncommon and cases presenting at birth or within a few days after birth are classified as congenital. CML is divided into three phases: chronic, accelerated, and blast phases (1). CML is defined as a myeloproliferative disease characterized by the presence of Philadelphia (Ph) chromosome or the BCR/ABL fusion gene. Although most patients could easily be diagnosed by peripheral blood evaluation, genetic studies are essential for the confirmation of the diagnosis. The WHO classification of CML is defined not only by its classic morphology and clinical features, but also by its genetic abnormality, the Ph chromosome, or the BCR/ABL fusion gene (2, 3).

Cranial nerve palsy is an uncommon presenting feature of leukemia and facial nerve palsy is rare in childhood (4, 5). The incidence of facial paralysis is 2.7 per 100000 in children under the age of 10 years. Facial palsy can be either congenital or acquired. Causes of acquired facial paralysis include trauma, infectious, inflammatory or metabolic diseases, vascular abnormality, and leukemic infiltration (5). Leukemic infiltrations of the CNS causing facial paralysis are extremely rare (4).

In this paper, we report an infant with symptoms of increased intracranial pressure affecting seventh cranial nerve. After an extensive investigation, involvement of the cranial nerve palsy in the setting of CML was identified. This is the first report of cranial neuropathy and leukemic infiltration involving the central nervous system as an initial manifestation of CML during childhood.

## Case report

A 1-month-old female with unilateral left facial palsy was referred to the Pediatric Clinic, Ali-bin-Abitaleb Hospital, Zahedan, eastern Iran in 2013. Left facial nerve palsy developed on the 13^th^ day after birth that she could not close her eye associated with loss of tearing from the affected eye. She had low-grade fever and purulent otorrhea developed 7 and 3 d prior to admission, respectively. There was no history of pregnancy complications and abnormal delivery. The patient was a full-term baby with normal vaginal delivery. At first assessment, she was alert but irritable, ill-appearing, and hypotonic. She was febrile with respiratory distress. Lymphadenopathy and organomegaly were not detected on physical examination. She had facial asymmetry and unilateral peripheral facial nerve palsy. Other parts of neurologic examination were normal. Bilateral tympanic membranes were hyperemic and showed signs of bulging. 

Informed consent was taken from the parents before reporting the case.

**Fig 1:**
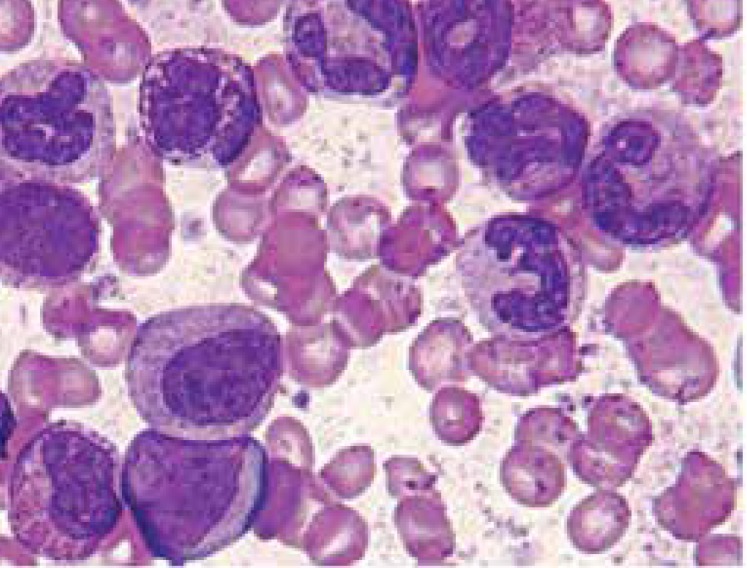
Peipheral blood smear

Initial laboratory evaluation was conducted which generated the following results: WBC: 157000/mm^3^, Hb: 6.2 gr/dL, Hct: 18.6%, and platelet: 130000/mm^3^. Analysis of CSF showed the followings: 670 RBC/dL, 630 WBC/dL (70% neutrophils, 30% lymphocytes), protein 14 mg/dL, and glucose 18 mg/dL. CRP was 1+; ESR was 70 mm/h. Cell morphology of her peripheral blood smear (PBS) showed polymorphonuclears (PMNs) and myelocytes were prominent rather than other cell types (Figure 1). Basophil count was approximately 20% of the total cells. Large platelets and mild granulocytic dysplasia were visible on peripheral blood smear.

Bone marrow aspiration and biopsy was performed. The flow cytometry results were as follows: HLA DR: 2%, CD2: 3.3%, CD5: 2.8%, CD7: 2.1%, CD10: 1.3%, CD13: 86.7%, CD14: 64.8%, CD19: 1.7%, CD20: 4.7%, CD33: 37%%, CD34: 3.4%, CD64: 97.4%, and CD117: 0.8%. Cytogenetic analysis revealed the presence of the Ph chromosome, which was suggestive of the BCR-ABL fusion gene. A diagnosis of CML was ascertained for the patient.

Brain CT scan did not reveal any intracranial abnormalities or evidence of mastoiditis. Vancomycin and ceftriaxone were initiated with oxygen therapy; however, no response to this treatment was observed. On the second day after admission, the patient developed a seizure. After 4 d of hospitalization, her left facial paralysis improved. On the 6^th^ day of hospitalization, a bone survey was conducted, which did not show any lesions or abnormalities throughout the bones. On ophthalmologic examination, opacity in the left iris was detected. On the 7^th^ day of hospitalization, the patient developed a right submandibular mass. 

**Fig 2:**
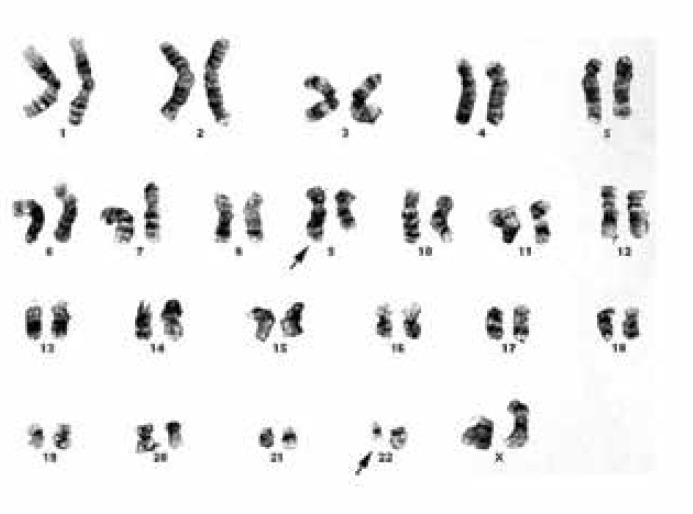
Chromosomal karyotype shows t (9, 22

Treatment with **GLEEVEC® (imatinib mesylate) **was started for the patient. After 12 d of hospitalization, the patient experienced cardiopulmonary arrest for which resuscitation was unsuccessful and the patient succumbed to death.

## Discussion

The most common causes of increased intracranial pressure in a pediatric patient include severe traumatic brain injury, hydrocephalus, brain tumor, infections (i.e., meningitis, encephalitis), metabolic encephalopathy, hypoxic-ischemic brain injury, cerebral infarction, and intracranial hemorrhage due to rupture of an arteriovenous malformation or aneurysm (6).

In this patient, increased intracranial pressure was due to central nervous system (CNS) infiltration of CML. Although CML accounts for about 20% of newly diagnosed cases of leukemia in adults (7), CML in children is rare and accounts for less than 10% of all CML cases and constitutes less than 2% of all pediatric leukemia. Due to the rarity of the disease, a few studies have been performed on this disease in children (8).

The occurrence of cranial neuropathies in children, especially involving seventh nerves, as a symptom of leukemic infiltration, is extremely rare. Facial palsy in lymphoid malignancies has been reported, which showed that approximately 45 out of 1000 children with acute lymphoblastic leukemia and non-Hodgkin’s lymphoma develop cranial nerve palsy. Eleven children with AML who had facial paralysis have also been reported. Cranial neuropathy as a manifestation of CML has not been reported in adults and children until recently (4). 

**In conclusion, **this is the first report of a child with CML who developed cranial neuropathy as leukemic infiltration.
